# Neurological symptoms in Covid‐19 patients in the emergency department

**DOI:** 10.1002/brb3.2058

**Published:** 2021-02-22

**Authors:** David García‐Azorín, Javier Trigo, Enrique Martínez‐Pías, Isabel Hernández‐Pérez, Gonzalo Valle‐Peñacoba, Blanca Talavera, Paula Simón‐Campo, Mercedes de Lera, Alba Chavarría‐Miranda, Cristina López‐Sanz, María Gutiérrez‐Sánchez, Elena Martínez‐Velasco, María Pedraza, Álvaro Sierra, Beatriz Gómez‐Vicente, Ángel Guerrero, Juan Francisco Arenillas

**Affiliations:** ^1^ Department of Neurology. Hospital Clínico Universitario de Valladolid Valladolid Spain

**Keywords:** COVID‐19, mortality, nervous system diseases, olfaction disorders, prognosis

## Abstract

**Background:**

Coronavirus disease 2019 (Covid‐19) might present neurological symptoms. We aimed to evaluate the frequency of them at the moment of emergency department (ED) visit and their impact in the prognosis.

**Methods:**

Retrospective cohort study including all consecutive hospitalized cases between March 8th and April 11th, 2020. Covid‐19 diagnosis was confirmed by polymerase chain reaction test and/or serology. We compared, in patients with and without neurological symptoms on admission, demographic, clinical presentation, and frequency and type of abnormal laboratory values. We analyzed the variables that were associated with in‐hospital all‐cause mortality by Cox‐regression log‐rank test.

**Results:**

We included 576 hospitalized patients, 250 (43.3%) female, aged 67.2 years. At the moment of ED visit, 320 (55.6%) described neurological symptoms, including anosmia (146, 25.3%), myalgia (139, 24.1%), headache (137, 23.8%), and altered mental status (98, 17.0%). Neurological symptoms started the first symptomatic day in 198 (54.2%) cases. Patients with neurological symptoms presented later to the ED (7.9 versus. 6.6 days, *p* = .019). Only four (0.6%) cases had no typical Covid‐19 general symptoms, and only six (1.9%) had a normal laboratory results, for a sensitivity of 98.7% (95% confidence interval (CI): 96.6%–99.6%) and 98.1% (95% CI: 95.7%–99.2%), respectively. In the multivariate Cox‐regression of mortality predictors, anosmia (HR: 0.358, 95%CI: 0.140–0.916) and altered mental status (HR: 1.867, 95%CI: 1.162–3.001) were significant.

**Conclusion:**

Neurological symptoms were the most frequent extrapulmonary symptoms. They were present in half of the Covid‐19 patients at the time of the ED visit. Anosmia on admission was an independent predictor of lower in‐hospital mortality and altered mental status on admission predicted in‐hospital mortality.

## Graphical abstract

Half of the Covid‐19 patients described neurological symptoms at the moment of ED presentation. The presence of neurological symptoms at the moment of ED presentation was an independent predictor of mortality. Patients with altered mental status died more and earlier, and patients with anosmia had a lower risk of mortality.

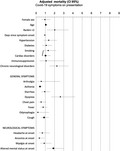

## 1 INTRODUCTION

The Severe Acute Respiratory Syndrome coronavirus 2 (SARS‐CoV‐2) is an RNA virus from the order Nidoviridae (Cui et al., 2019; Zhou, Yang, et al., 2020). The clinical presentation ranges from a mild upper respiratory tract infection to a severe pneumonia with acute distress respiratory syndrome (ADRS) (Zhou, Yang, et al., 2020), causing death to 8.0% of patients in Spain (National Epidemiologic Surveillance Network, 2020). Besides the respiratory symptoms, the disease might compromise other organs, causing acute kidney injury, acute cardiac injury, arrhythmia, disseminated intravascular coagulopathy, or shock (Chang et al., 2020; Chen et al., 2020; Guan et al., 2020; Huang et al., 2020; Wang et al., 2020; Xu et al., 2020).

The frequency, type, and onset of neurological symptoms is largely unexplored. Since the initial series of cases, neurological symptoms are the most frequent extrapulmonary manifestations (Chang et al., 2020; Chen et al., 2020; Guan et al., 2020; Huang et al., 2020; Wang et al., 2020; Xu et al., 2020). Frequency of myalgia is 20.4% in mean (range: 11%–44%) (Chang et al., 2020; Chen et al., 2020; Guan et al., 2020; Huang et al., 2020; Wang et al., 2020; Wu et al., 2019; Xu et al., 2020), headache is described in 13.6% of patients (range: 4%–24%) (Chang et al., 2020; Chen et al., 2020; Guan et al., 2020; Huang et al., 2020; Shi et al., 2020; Wang et al., 2020; Xu et al., 2020), and anosmia is described in 5%–88% of patients (Angelo Vaira et al., 2020; Beltrán‐Corbellini et al., 2020; Helms et al., 2020; Lechien et al., 2020). The few studies that specifically analyzed the frequency of neurological symptoms reported a frequency of 84% in critically ill patients (Mao et al., 2019) and 36.4% in hospitalized patients (Helms et al., 2020). Some studies describe that neurological symptoms are more frequent in severe patients (Helms et al., 2020; Mao et al., 2019), but the real impact on prognosis is unclear (Herman et al., 2020).

The aims of the present study were (1) to evaluate the frequency and type of neurological symptoms at the moment of ED presentation; (2) to analyze the moment when the neurological symptoms started during the course of Covid‐19 disease; (3) to study if the presence of Covid‐19 in patients that present to the ED with neurological symptoms can be suspected based on the presence of general Covid‐19 symptoms and/or laboratory abnormalities; and (4) to evaluate the association between the presence of neurological symptoms and the prognosis of Covid‐19, evaluated as in‐hospital, all‐cause mortality.

## 2 METHODS

This is an observational study with retrospective cohort design. The study was done according to the strengthening to the reporting in observational studies in epidemiology (STROBE) guidelines (Elm et al., 2007). The study population included patient with confirmed Covid‐19 disease that required hospitalization. The study was done in the Hospital Clínico Universitario de Valladolid, Valladolid, Spain, a tertiary public hospital with a reference population of 280.000 inhabitants. The study was approved by the local ethics review board (PI‐20–1751), which waived the need of written informed consent. The study was done in accordance with the Declaration of Helsinki principles. The study period included patients admitted from March 8th to April 11th, with a follow‐up up to May 1st, 2020, including a minimum of 20 days of follow‐up after admission in all patients.

### 2.1 Eligibility criteria

Patients were included if they 1) had confirmed Covid‐19 disease; 2) were hospitalized; and 3) were aged 18 or older. They were excluded if the hospitalization was not done from the emergency department or if there was not any available information about the hospitalization period. The sampling was nonprobabilistic, and all the consecutive confirmed cases were recruited. By the time when the study was done, due to the collapse of the healthcare system, patients with a poor medical condition or those residents of nursing homes were instructed to receive the treatment at home, whenever indicated.

### 2.2 Diagnosis of Covid‐19 disease

Covid‐19 diagnosis was based on real‐time reverse‐transcriptase‐polymerase‐chain‐reaction (RT‐PCR) assay (LightMIx Modular SARS‐CoV (COVID19) E‐gene and LightMIx Modular SARS‐CoV (COVID19) RdRP, Roche Diagnostics S.L.) from oropharyngeal‐nasopharyngeal swab, sputum, or lower respiratory tract sample; or by the presence of anti‐SARS‐CoV‐2 IgM + IgA antibodies (COVID‐19 ELISA IgM + IgA; Vircell, S.L. Granada, Spain) in serological test in patients with clinical symptoms, according to the World Health Organization (WHO) protocols (World Health Organization,).

### 2.3 Data sources

We collected data from electronic medical records from primary care, emergency department, and hospitalization. According to the local protocol, patients were instructed to contact a specific Covid‐19 line that followed‐up them daily or every other day before and after the hospitalization until decease or curation. In the emergency department, every patient fulfilled a symptom checklist that included anosmia, headache, myalgia, or syncope among other symptoms. During the hospitalization, patients were treated according to the national Covid‐19 management protocol standard of care (SOC) (Ministry of Health, 2020; World Health Organization, 2020). We reviewed the daily reports from the indicated sources preceding the admission, and during and after the hospitalization period. All the data were gathered by neurologists that belonged to the Covid‐19 multidisciplinary teams.

### 2.4 Variables

The demographic variables included age, sex, the date of general symptoms onset, the date of neurological symptoms onset, and the suspected source of contagion. The prior medical history data included vascular risk factors, including hypertension (systemic blood pressure higher than 140/90 mmHg in two prior determinations), diabetes (fasting blood glucose > 126 mg/dl on two separate tests, HbA1c > 6.5%, blood glucose level > 200 mg after oral glucose overload or blood glucose level > 200 mg/dl with diabetes symptoms), and smoking habit (current or in the preceding 6 months). We assessed the presence of chronic comorbid conditions, including cardiovascular diseases (coronary artery disease, congenital heart diseases, cardiomyopathies, arrythmias, valvular heart disease, aortic aneurysms, and peripheral artery disease), chronic pulmonary diseases (chronic obstructive pulmonary disease (COPD), asthma, occupational lung diseases, interstitial lung diseases, and pulmonary hypertension), cancer (excluding epidermoid and basal cell carcinoma), immunocompromised state (congenital or acquired), and chronic neurological disorders (neurological disorders causing persistent disability, impairing the individual's functioning and with interference with the person's ability to engage in activities). The baseline performance of patients was determined by the modified Rankin scale, and we used three points as threshold of disability (Quinn et al., 2009).

We systematically analyzed the presence of neurological symptoms, including anosmia/hyposmia, altered mental status (Smith & Han, 2019), headache, myalgia, syncope, seizures, acute sudden focal symptoms (paresis, hypoesthesia, ataxia, and speech disorders), vertigo, and others. We focused on symptoms because at the moment of ED presentation, the cause of the symptoms may not be clear yet. We considered all possible neurological symptoms as described in other series, regardless that some of them (e.g., myalgia) can be considered as general symptoms as well. Frequency of type of general symptoms was also systematically screened, including arthralgia, asthenia, weakness, diarrhea, dyspnea, chest pain, expectoration, fever, light‐headedness, odynophagia, cutaneous rash, rhinorrhea, cough, and vomiting.

We assessed the frequency of abnormal laboratory examinations on admission, including leukocytes (reference value (RV): 4–10 cell count x 109 / L), the presence of lymphopenia (<0.9 cell count x 109 / L), the presence of anemia (< 12 g / dL), platelets (RV: 150–400 count x 10^9^ / L), increased lactate dehydrogenase (LDH) level (>250 U / L), decreased glomerular filtration rate (GFR) (<90 ml/min/1.73m^2^), abnormal liver enzymes, including aspartate aminotransferase (AST) (RV < 32 U / L), alanine aminotransferase (ALT) (RV < 33 U / L), and gamma‐glutamyl transferase (GGT) (RV < 40 U / L); increased creatine‐kinase (CPK) (>170 U / L), increased international normalized ratio (INR) (>1.3), D‐dimer (RV < 500 ng / dL), increased C‐reactive protein (CRP) (> 5 mg / L), and increased procalcitonin (PCT) (> 5 ng / mL).

The severity of the disease was categorized according to the American Thoracic Society guidelines for community‐acquired pneumonia (Metlay et al., 2019) into mild disease, pneumonia, severe pneumonia, and acute distress respiratory syndrome (ADRS). The specific definitions are available in the supplementary materials (Supplementary Table 1).

### 2.5 Cohort description

The study cohort included all the consecutive patients that were hospitalized and had a confirmed Covid‐19 diagnosis, according to the eligibility criteria. To answer the research questions, we reviewed, in the abovementioned sources, the frequency, onset and type of neurological symptoms. Due to the exceptional sociosanitary circumstances caused by Covid‐19, the urgent need of data, and the noninterventional nature of the project, patients were not involved neither in the design of the study nor in the recruitment of the study. The information about patients was codified by investigators before including it into the database.

### 2.6 Patient and public involvement statement

Due to the socio‐sanitary situation, patients were not involved in the design of the study. The present work was performed without any direct or indirect support from public entities. The study was designed by the authors, who collected and analyzed the data, and wrote the manuscript.

### 2.7 Statistical analysis

We present qualitative variables as frequency and percentage. Quantitative variables are presented as mean and standard deviation (*SD*) or median and interquartile range (IQR). We analyzed the association between qualitative variables with the Chi‐square test, using the Bonferroni method in the multiple comparisons correction, presenting directly the adjusted p‐value. In the contrast of qualitative and quantitative variables, we used the Student *t* test or the Mann–Whitney U test if the distribution was not normal according to the Kolmogorov–Smirnov test. The statistical signification threshold was 0.05, after adjusting for multiple comparisons. We did a post‐hoc subanalysis in which we compared the frequency of symptoms in patients with nonspecific neurological symptoms (excluding patients with headache, myalgia, and anosmia) on presentation, compared with the rest of the sample, as well.

For the third study aim, assessing whether the presence of Covid‐19 could be suspected in patients presenting to the ED with neurological symptoms, we calculated the sensitivity of each general symptom and each laboratory parameter. Sensitivity was defined as the number of patients with an altered laboratory parameter or a general symptom present over the total of patients with the disease. We present it together with the 95% confidence interval (CI). Finally, we analyzed the association with a higher mortality with Cox‐regression log‐rank test and logistic regression. We did a univariate regression, and all the variables that had an alpha error equal or lower than 20% were included in the multivariate regression analysis. We describe the hazard ratio (HR), odds ratio (OR), and 95% CI. To minimize the overfitting of the model, we did a sensitivity analysis, and the model was repeated including those variables with an alpha error equal or lower than 10%. Sample size was not calculated in advance, but we estimated the power of our study to be of 95%. The calculation is available in supplementary materials (supplementary files). We analyzed if missing data were completely at random and when it was the case and the percentage of missing data was < 5%, we used complete case analysis. If it was not completely at random, we used worst‐case scenario carried forward imputation or we assumed that the variable was not abnormal. Statistical analysis was done with SPSS v.26 (IBM Corp. Armonk, NY).

## 3 RESULTS

During the study period, 580 patients were screened and 576 were included in the study. Three patients were excluded because they were not admitted from the emergency department and one patient because there was no available information. The mean age of the sample was 67.2 (*SD*: 14.7) years, and 250 (43.4%) patients were female. In 320 (55.6%) patients, one or more neurological symptoms were described on admission. Patients with neurological symptoms were younger, with better baseline performance, and with fewer prior cardiac disorders. Table 1 shows the demographic variables, the frequency of vascular risk factors, and comorbidities.

**TABLE 1:** Demographic variables, frequency of vascular risk factors, and frequency of comorbidities in the whole sample and within the groups of patients with and without neurological symptoms on admission

Variable	All patients (*n* = 576)	Neurological symptoms on admission (*n* = 320)	No neurological symptoms on admission (*n* = 256)	Adjusted p‐value
Female sex (frequency and %)	250 (43.4%)	150 (46.9%)	100 (39.1%)	0.073
Age (years)	67.19 (14.75)	64.91 (14.11)	70.03 (15.07)	<0.001
Modified Rankin scale (mean score)	0.61 (1.12)	0.48 (1.02)	0.77 (1.21)	0.002
Hypertension (frequency and %)	300 (52.1%)	159 (49.7%)	141 (55.1%)	0.229
Diabetes (frequency and %)	113 (19.6%)	65 (20.3%)	48 (18.8%)	0.716
Smoking (frequency and %)	118 (20.5%)	56 (17.5%)	62 (24.2%)	0.060
Cardiac disorders (frequency and %)	154 (26.7%)	73 (22.8%)	81 (31.6%)	0.022
Pulmonary disorders (frequency and %)	145 (25.2%)	75 (23.4%)	70 (27.3%)	0.329
Cancer (frequency and %)	94 (16.3%)	47 (14.7%)	47 (18.4%)	0.284
Immunosuppression (frequency and %)	32 (5.6%)	13 (4.1%)	19 (7.4%)	0.117
Chronic neurological disorders (frequency and %)	105 (18.2%)	51 (15.9%)	54 (21.1%)	0.138

The presumptive source of the contagion was attributed to a close relative in 96 (16.6%) cases, occurred in a retirement home with other positive cases in 64 (11.1%), was related with a journey to a geographical area with infected people in 39 cases (6.7%), was related with healthcare work in 23 cases (3.9%), was related with nonhealthcare work in 21 (3.6%), was nosocomial in 5 (0.8%), and the source was unknown in 328 (56.9%). In patients with neurological symptoms at onset, frequency of unknown source of contagion was 161 (50.3%) compared with 133 (52.0%) in those without neurological symptoms (*p* = .758). Diagnosis was confirmed by PCR in 546 (94.8%) of cases and serology in 175 (30.4%). Chest imaging was abnormal in 549 (95.3%) patients.

### 3.1 Frequency and type of neurological symptoms

Of 576 patients, 374 (64.9%) had neurological symptoms at any point at presentation and admission. Of the 374 patients, 320 (85.6%) cases presented with neurological symptoms in the ED. The most frequent neurological symptoms at the moment of ED presentation were anosmia, in 146 (25.3%) patients, myalgia in 139 (24.1%), headache in 137 (23.8%), and altered mental status in 98 (17.0%). All the neurological symptoms were described more frequently on admission than during hospitalization except for stroke, seizures, and ataxia. Table 2 describes the relative frequency of neurological symptoms on admission and during hospitalization.

**TABLE 2:** Frequency and type of neurological symptoms on admission and during hospitalization

Variable	Frequency in the whole sample (*n* = 576)	Neurological symptoms on admission (*n* = 320)	No neurological symptoms on admission (*n* = 256)	Adjusted p‐value
Headache	137 (23.8%)	124 (90.5%)	13 (9.5%)	<0.001
Anosmia	146 (25.3%)	133 (91.1%)	13 (8.9%)	<0.001
Myalgia	139 (24.1%)	129 (92.8%)	10 (7.2%)	<0.001
Altered mental status	98 (17.0%)	70 (71.4%)	28 (28.6%)	0.001
Sudden focal symptoms	12 (2.1%)	7 (58.4%)	5 (41.7%)	1.000
Seizures	3 (0.5%)	2 (66.7%)	1 (33.3%)	1.000
Ataxia	6 (1.0%)	5 (83.3%)	1 (16.7%)	0.335
Weakness	14 (2.4%)	6 (42.9%)	8 (57.1%)	0.482
Syncope	43 (7.5%)	37 (86.0%)	6 (14.0%)	<0.001
Vertigo	11 (1.9%)	10 (90.9%)	1 (9.1%)	0.038

### 3.2 Onset of neurological symptoms

**FIGURE 1:**
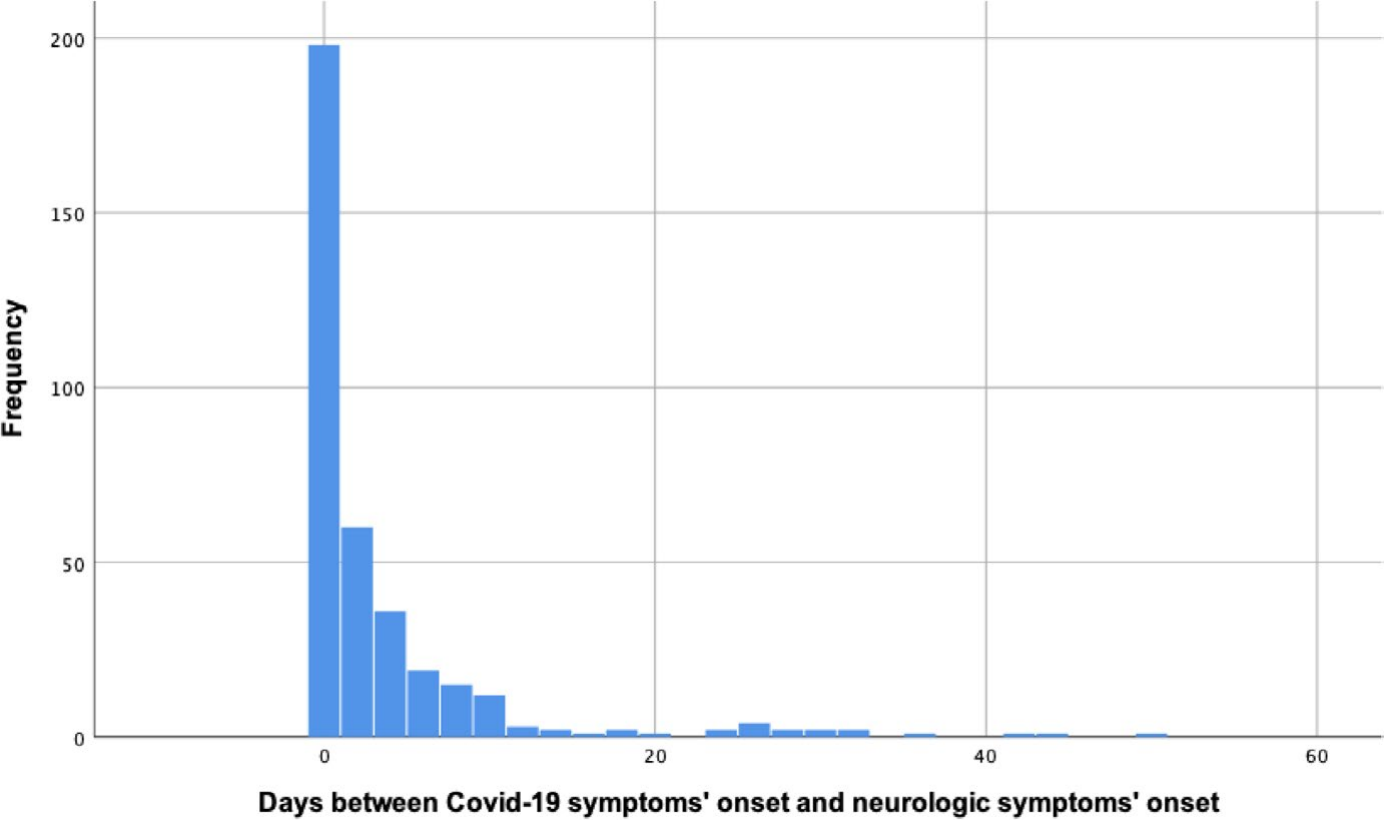
Interval between the onset of Covid‐19 general symptoms and neurological symptoms onset

**FIGURE 2:**
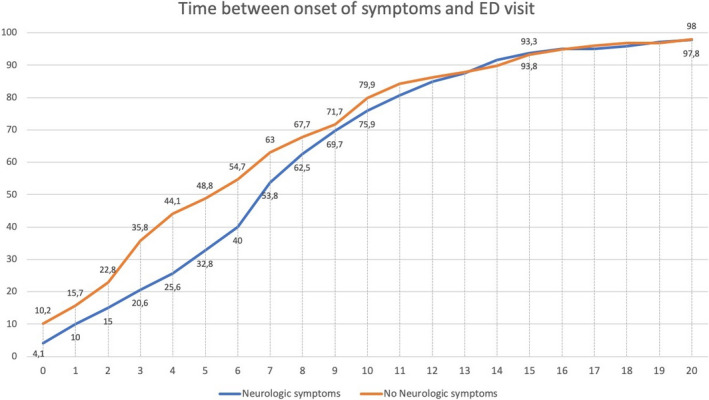
Cumulative percentage of patients that had visited the emergency department each day since the symptoms’ onset. The blue line represents patients with neurological symptoms and the orange line patients without neurological symptoms

Neurological symptoms started the same day as the rest of the Covid‐19 symptoms in 198 (54.2% of all patients with neurological symptoms on presentation) patients. By the fourth day of symptoms, neurological symptoms were present in 294 (80.5%) of patients. Figure 1 shows the interval between the onset of Covid‐19 general symptoms and neurological symptoms. Patients with neurological symptoms came to the ED after 7.9 (*SD*: 5.5) days, compared with 6.6 (*SD*: 6.9) in those without (*p* = .019). Figure 2 shows the cumulative percentage of patients that had visited the ED each day since the symptoms’ onset.

### 3.3 Frequency of general symptoms

Patients with neurological symptoms on admission had more frequently arthralgia, asthenia, fever, light‐headedness, rash, and cough than those without it. Table 3 shows the relative frequency of Covid‐19 symptoms, and supplementary Table 2, the same results in patients with nonspecific neurological symptoms. After adjusting for sex, age, baseline performance, and days since the onset symptoms, patients with neurological symptoms on admission had higher odds of having arthralgia (OR: 12.29, 95% CI: 2.9–52.11, *p* = .001), and light‐headedness (OR: 3.25, 95% CI: 1.68–6.26, *p*=<0.001), but not asthenia (OR: 1.31, 95% CI: 0.92–1.86, *p* = .129), fever (OR: 1.37, 95% CI: 0.89–2.12, *p* = .151), rash (OR: 6.89, 95% CI: 0.86–54.93, *p* = .069), or cough (OR: 1.36, 95% CI: 0.93–2.00, *p* = .109).

**TABLE 3:** Type, frequency, and percentage of Covid‐19 symptoms

Variable	All patients (*n* = 576)	Neurological symptoms on admission (*n* = 320)	No neurological symptoms on admission (*n* = 256)	Adjusted p‐value
Arthralgia	35 (6.1%)	33 (10.3%)	2 (0.8%)	<0.001
Asthenia	242 (42.1%)	148 (46.3%)	94 (36.7%)	0.027
Weakness	90 (15.7%)	58 (18.1%)	32 (12.5%)	0.083
Diarrhea	192 (33.4%)	113 (35.3%)	79 (30.9%)	0.299
Dyspnea	292 (50.8%)	161 (50.3%)	131 (51.2%)	0.904
Chest pain	99 (17.2%)	58 (18.1%)	41 (16.1%)	0.593
Expectoration	90 (15.7%)	56 (17.5%)	34 (37.8%)	0.204
Fever	462 (80.3%)	268 (83.8%)	194 (75.8%)	0.023
Light‐headedness	60 (10.4%)	47 (14.7%)	13 (5.1%)	<0.001
Odynophagia	60 (10.4%)	36 (11.3%)	24 (9.4%)	0.552
Cutaneous rash	11 (1.9%)	10 (3.1%)	1 (0.4%)	0.038
Rhinorrhea	12 (2.1%)	6 (1.9%)	6 (2.3%)	0.922
Cough	403 (70.2%)	240 (75.0%)	163 (63.9%)	0.005
Vomiting	47 (8.2%)	25 (7.8%)	22 (8.6%)	0.852

The presence of general Covid‐19 symptoms in patients presenting with neurological symptoms had a 98.7% (96.6%–99.6%) sensitivity. There were four (0.6%) patients that presented with neurological symptoms and did not have any Covid‐19 symptom on admission (Supplementary Table 3). Three patients consulted because of loss of consciousness, and the other case consulted because of delirium. The symptoms with higher sensitivity were fever (83.7%), cough (75%), and dyspnea (50.3%). Supplementary Table 4 shows the sensitivity of general symptoms in Covid‐19 patients with neurological symptoms on admission.

### 3.4 Laboratory parameters

The frequency of laboratory abnormalities was similar in patients with and without neurological symptoms on admission except for lactate dehydrogenase (187 (58.4%) versus. 175 (68.4%), *p* = .018) and glomerular filtration rate (201 (62.8%) versus. 185 (72.3%), *p* = .021), which were both more frequently abnormal in patients without neurological symptoms on admission. Supplementary Table 5 shows the frequency of laboratory abnormalities in the sample and within groups.

The presence of laboratory abnormalities in patients presenting with neurological symptoms had a 98.1% (95.7%–99.2%). There were six (1.9%) patients with fully normal laboratory parameters on admission. The combination of lymphopenia, and increased LDH, INR, D‐dimer, CRP, and procalcitonin had a 96.9% (94.1%–98.4%) sensitivity. All the patients with normal laboratory parameters had Covid‐19 symptoms, being the sensitivity of combination of clinical symptoms and laboratory parameters of 100% (99.1%–100%). In patients with neurological symptoms, the laboratory parameters with the higher sensitivity were C‐reactive protein (88.7%), D‐dimer (65.3%), and GFR (62.8%). Supplementary Table 6 shows the sensitivity of laboratory parameters in Covid‐19 patients with neurological symptoms on admission. Supplementary Figure 1 shows the sensitivity of the general symptoms and laboratory abnormalities in Covid‐19 patients presenting with neurological symptoms.

### 3.5 Predictors of mortality

The course of the disease corresponded to mild disease in 32 (5.6%) patients, pneumonia in 142 (24.7%), severe pneumonia in 269 (46.8%), and ADRS in 124 (21.6%). All‐cause in‐hospital mortality rate was 127/576 (22.0%), being lower in patients without neurological symptoms on admission (58/320, 18.1% versus. 69/256, 27.0%, *p* = .015). Age, mRS, time since symptoms´ onset, hypertension, diabetes, smoking habit, prior history of cardiac disorders, chronic neurological disorders, and immunosuppression were associated with higher odds of mortality. The presence of headache, anosmia, myalgia, asthenia, diarrhea, chest pain, fever, odynophagia, and cough on admission were associated with a lower odds of death, and the presence of altered mental status or dyspnea on admission were associated with higher mortality in the univariate analysis. Supplementary Table 7 shows the results of the univariate Cox‐regression analysis, and supplementary Table 8 presents the results of logistic regression analysis.

**FIGURE 3:**
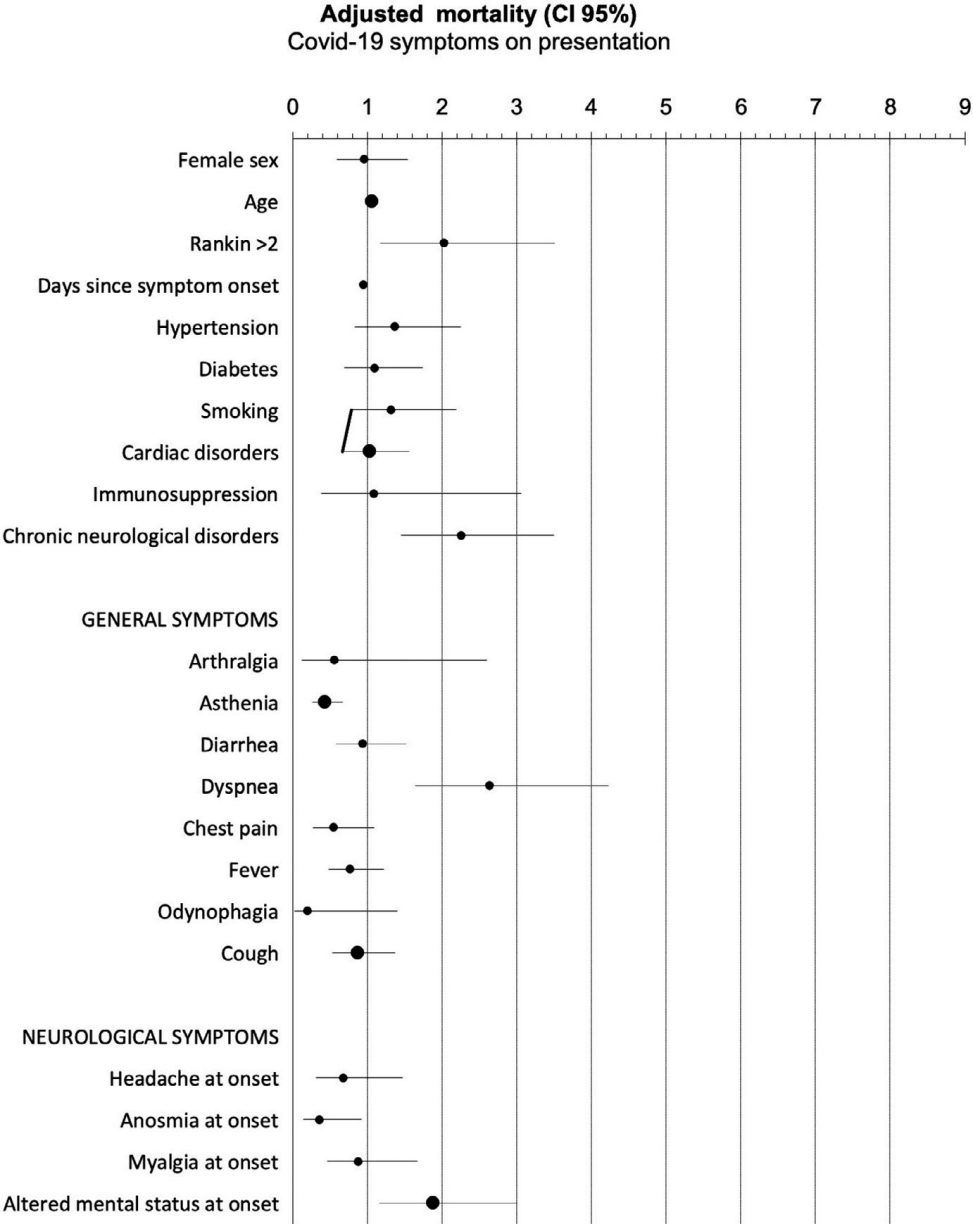
Results of the multivariate Cox‐regression analysis: Hazard ratio and 95% confidence interval

In the multivariate Cox‐regression analysis, the variables that were associated with a higher odd of death were age (HR: 1.047, 95% CI: 1.022–1.072), mRS (HR: 2.029, 95%CI: 1.172–3.512), chronic neurological disorders (HR: 2.250, 95%CI: 1.446–3.502), altered mental status (HR: 1.867, 95%CI: 1.162–3.001), and dyspnea (HR: 2.636, 95%CI: 1.642–4.231); while time since symptoms onset (HR: 0.946, 95%CI: 0.913–0.981), the presence of anosmia (HR: 0.358, 95%CI: 0.140–0.916), and asthenia (HR: 0.420, 95%CI: 0.262–0.671) were statistically significant. The restrictive analysis did not alter the results. Supplementary Tables 9–12 present the detailed results of the multivariate Cox‐regression and logistic regression. Figure 3 shows the results of the multivariate Cox‐regression analysis.

## 4 DISCUSSION

In the present study, neurological symptoms were among the most frequent clinical manifestations of Covid‐19, occurring in half of the patients. Only fever (80%), cough (70%), dyspnea (50%), and asthenia (40%) were more frequent than neurological symptoms such as anosmia (25%), myalgia (24%), and headache (23%). It is not widely recognized that Covid‐19 might present with neurological symptoms, as some official guidelines (Ministry of Health, 2020) do not include them within the typical manifestations. This could explain the delay in ED presentation of patients that had neurological symptoms at onset. Since specific Covid‐19 tests may not be available everywhere, or may consume if the supply is not granted, we aimed to evaluate if the presence of Covid‐19 in patients presenting to the ED could be suspected based on the presence of the clinical presentation and the laboratory results.

Besides frequent, most of the neurological symptoms had an early presentation. More than half of the patients with neurological symptoms had them since the first symptomatic day and 80% of the patients by the fourth day. This should raise the awareness of general practitioners, emergency department physicians, and neurologists that Covid‐19 might present with unspecific neurological symptoms (Asadi‐Pooya & Simani, 2020; Herman et al., 2020). Headache and myalgia are common in other viral illnesses. Anosmia is associated with some viral infections, such as rhinovirus, picornavirus, parainfluenza, Epstein‐Barr virus, or other human coronaviruses (Suzuki et al., 2007). Altered mental status could be related with the presence of hypercapnia, acute kidney injury, acute liver failure, fever, drugs, or even the viremia (Smith & Han, 2019; Ward et al., 2020). In our sample, it seemed to predict a more severe presentation, as it was an independent predictor of mortality (Ward et al., 2020).

Most of the patients present general symptoms as well (Chang et al., 2020; Chen et al., 2020; Guan et al., 2020; Huang et al., 2020; Wang et al., 2020; Xu et al., 2020). In our sample, less than one percent of the patients not had any systemic symptom at the time of the ED presentation, and all those patients had typical laboratory abnormalities. There is not a perfect biomarker yet, as neither the clinical symptoms nor the laboratory parameters have a perfect sensitivity (World Health Organization,). During the pandemic, presence of Covid‐19 should be considered in every patient, and the work‐up should include laboratory determinations and clinical symptoms, including neurological symptoms, which are mainly unspecific, being the most frequent anosmia, myalgia, and headache (Asadi‐Pooya & Simani, 2020).

Apart from the relevance in the diagnosis, one of the most striking findings of this study was the impact of the neurological symptoms in the prognosis of patients. We analyzed the association between the presence of neurological symptoms on presentation, and after adjusting for age, sex, baseline performance, time since the symptoms onset, vascular risk factors, comorbidities, and general symptoms, anosmia and altered mental status were still associated with a lower and higher odd of mortality, respectively. Prior studies described the association of age, vascular risk factors, comorbidities, and analytic parameters (Chang et al., 2020; Chen et al., 2020; Guan et al., 2020; Huang et al., 2020; Ruan et al., 2020; Wang et al., 2020; Wu et al., 2019; Xu et al., 2020; Zhou, Yu, et al., 2020), but not the presence of neurological symptoms on presentation. In the present study, some relevant comorbidities, as diabetes or cardiac disorders were associated with a worse prognosis in the univariate models but not in the multivariate models. The high number of analyzed parameters could decrease the power of the study and some variables could be falsely negative. Patients with poor medical condition and severe comorbidities compromising the life expectancy may not be candidates for aggressive life‐sustaining therapies. However, at the peak of the first wave of the pandemic, most of those patients were not candidates to be hospitalized due to the collapse of the healthcare system and were managed in an outpatient basis.

The precise signification of each symptom should probably be analyzed separately. We did not differentiate the specific phenotype of the headache, while some reports suggest that different headaches might coexist (Belvis, 2020). The duration over time and association with other prognostic variables, other than mortality, should be explored. We focused on the clinical description and not in the pathophysiology or etiology of the manifestations. The neuroinvasive potential of the virus is yet uncertain (Mannan Baig et al., 2020), and despite the vast number of worldwide cases, the number of cases with confirmed cerebrospinal fluid isolation of SARS‐CoV‐2 is testimonial (Asadi‐Pooya & Simani, 2020; Herman et al., 2020). Many symptoms are relatively unspecific and may be present in patients with other viral infections. Studies assessing large samples of patients should clarify the role of the few neurospecific symptoms. In the case of anosmia (Talavera et al., 2020) or headache (Trigo et al., 2020), patients with those presented a different profile of general symptoms and a laboratory results, suggesting a different immune response, which could be partially responsible of the clinical presentation. In the case of altered mental status, as in other severe infections, in most cases, it seems to be associated with systemic complications, which are indeed associated with a higher risk of mortality.

Our study has some important flaws. It is a single‐center study, and the epidemiology and prognosis might not be representative of other settings. Multicentric, multinational studies are desirable in order to clarify the possible influence of genotype and ambient. Despite the data were collected in a systematic way, the retrospective design might underestimate the frequency of some clinical variables. Another relevant limitation is that follow‐up of the patients was limited to the first 20 days after admission, so we just analyzed in‐hospital mortality as some patients could have died afterward. We did not evaluate the specificity of general Covid‐19 symptoms or laboratory abnormalities, but future studies should consider to analyze that, which could clarity which of those has a better area under the curve. Further studies should consider mid and long‐term outcomes to see the impact of neurological symptoms over time.

## 5 CONCLUSION

Half of the Covid‐19 patients described neurological symptoms at the moment of ED presentation. The most frequent symptoms were anosmia, myalgia, and headache. Patients with neurological symptoms described them since the first day of Covid‐19 symptoms in 54% of cases and occurred by the fourth day of symptoms in 80% of cases. Patients with neurological symptoms waited 1.4 days more, in mean, before visiting the ED. Almost all the patients with neurological symptoms associated general typical Covid‐19 symptoms and, or laboratory abnormalities.

The presence of neurological symptoms at the moment of ED presentation was an independent predictor of mortality. Patients with altered mental status died more and earlier, and patients with anosmia had a lower risk of mortality.

## CONFLICT OF INTEREST

Authors declare no conflict of interest. The present study was performed without any direct or indirect financial support. East Valladolid Ethics Review Board approved the study (PI‐20–1751). All the datasheets are available for other authors upon reasonable request, by emailing the corresponding author (davilink@hotmail.com).

## AUTHORS’ CONTRIBUTIONS

**Table:** 

Name	Contribution
David García‐Azorín	Designed and conceptualized the study; major role in the acquisition of data; analyzed the data; drafted the manuscript for intellectual content.
Javier Trigo	Major role in the acquisition of data, revised the manuscript of intellectual content.
Enrique Martínez‐Pías	Major role in the acquisition of data, revised the manuscript of intellectual content.
Isabel Hernández‐Pérez	Major role in the acquisition of data, revised the manuscript of intellectual content.
Gonzalo Valle‐Peñacoba	Major role in the acquisition of data, revised the manuscript of intellectual content.
Blanca Talavera	Major role in the acquisition of data, revised the manuscript of intellectual content.
Paula Simón‐Campo	Major role in the acquisition of data, revised the manuscript of intellectual content.
Mercedes de Lera	Major role in the acquisition of data, revised the manuscript of intellectual content.
Alba Chavarría‐Miranda	Major role in the acquisition of data, revised the manuscript of intellectual content.
Cristina López‐Sanz	Major role in the acquisition of data, revised the manuscript of intellectual content.
María Gutiérrez‐Sánchez	Major role in the acquisition of data, revised the manuscript of intellectual content.
Elena Martínez‐Velasco	Major role in the acquisition of data, revised the manuscript of intellectual content.
María Pedraza	Major role in the acquisition of data, revised the manuscript of intellectual content.
Álvaro Sierra	Major role in the acquisition of data, revised the manuscript of intellectual content.
Beatriz Gómez‐Vicente	Major role in the acquisition of data, revised the manuscript of intellectual content.
Ángel Guerrero	Designed and conceptualized the study; analyzed the data; revised the manuscript for intellectual content.
Juan Francisco Arenillas	Designed and conceptualized the study; analyzed the data; revised the manuscript for intellectual content.

David García‐Azorín designed and conceptualized the study, played major role in the acquisition of data, analyzed the data, and drafted the manuscript for intellectual content. Javier Trigo, Enrique Martínez‐Pías, Isabel Hernández‐Pérez, Gonzalo Valle‐Peñacoba, Blanca Talavera, Paula Simón‐Campo, Mercedes de Lera, Alba Chavarría‐Miranda, Cristina López‐Sanz, María Gutiérrez‐Sánchez, Elena Martínez‐Velasco, María Pedraza, Álvaro Sierra, and Beatriz Gómez‐Vicente played major role in the acquisition of data and revised the manuscript of intellectual content. Ángel Guerrero and Juan Francisco Arenillas designed and conceptualized the study, analyzed the data, and revised the manuscript for intellectual content.

### PEER REVIEW

The peer review history for this article is available at https://publons.com/publon/10.1002/brb3.2058.

## Supporting information

Supplementary Material

## Data Availability

All the datasheets are available for other authors upon reasonable request, by emailing the corresponding author.
