# Supplementary material for: Neurological symptoms in Covid‐19 patients in the emergency department
Source: Brain Behav. 2021 Feb 22;11(4):e02058. doi: 10.1002/brb3.2058 (PMC7994975; doi:10.1002/brb3.2058)
Supplement: Supplementary file 1 — Supplementary Material [file BRB3-11-e02058-s001.docx]

**Supplementary materials:**

**Emergency department presentation of patients with Covid-19 disease with neurological symptoms: A cohort study on the clinical impact on 576 cases.**

David García-Azorín^1, 2^ MD, Javier Trigo^1^ MD, Enrique Martínez-Pías^1^ MD, Isabel Hernández-Pérez^1^ MD, Gonzalo Valle-Peñacoba^1^ MD, Blanca Talavera^1^ MD, Paula Simón-Campo^1^ MD, Mercedes de Lera^1^ MD, Alba Chavarría-Miranda^1^ MD, Cristina López-Sanz^1^ MD, María Gutiérrez-Sánchez^1^ MD, Elena Martínez-Velasco^1^ MD, María Pedraza^1^ MD, Álvaro Sierra^1^ MD, Beatriz Gómez-Vicente^1^ MD, Ángel Guerrero^1, 2,3^ MD, PhD, Juan Francisco Arenillas^1, 3, 4^ MD, PhD.

1. Department of Neurology. Hospital Clínico Universitario de Valladolid, Valladolid, Spain.
2. Institute for Biomedical Research of Salamanca (IBSAL), Salamanca, Spain.
3. Department of Medicine, University of Valladolid, Valladolid, Spain.

Neurovascular Research Laboratory. Instituto de Biología y Genética Molecular. Universidad de Valladolid – Consejo Superior de Investigaciones Científicas, Madrid.

**Corresponding author:**

David García-Azorín.

Department of Neurology, Hospital Clínico Universitario de Valladolid.

Avenida Ramón y Cajal n° 3, 47003 Valladolid. Spain.

Telf +34983420000 Ext. 87289.

E-mail address: [davilink@hotmail.com](mailto:davilink@hotmail.com), [dgazorin@ucm.es](mailto:dgazorin@ucm.es)

**Supplementary material index:**

1. Supplementary table 1: Severity of the Covid-19 disease
2. Sample size and power calculation.
3. Supplementary table 2: Frequency and type of neurological symptoms on admission and during hospitalization.
4. Supplementary table 4: Frequency of laboratory abnormalities in the whole sample and in patients with and without neurological symptoms on admission.
5. Supplementary table 5: Sensitivity of laboratory parameters in Covid-19 patients with neurological symptoms on admission.
6. Supplementary figure 1: Sensitivity (and 95% confidence interval) of general symptoms and laboratory parameters in Covid-19 patients with neurological symptoms on admission.
7. Supplementary table 6: Predictors of mortality: Univariate Cox-regression analysis.
8. Supplementary table 7: Predictors of mortality: Univariate logistic regression analysis.
9. Supplementary table 8: Predictors of mortality: Multivariate Cox-regression analysis.
10. Supplementary table 9: Predictors of mortality: Multivariate logistic regression analysis.

**Supplementary table 1:**

**Severity of the disease (Based on American Thoracic Society guidelines for community-acquired pneumonia^1^).**

| Severity level | Description |
| --- | --- |
| Mild illness | Patients with uncomplicated upper respiratory tract viral infection symptoms and have non-specific symptoms such as fever, fatigue, cough (with or without sputum production), anorexia, malaise, muscle pain, sore throat, dyspnea, nasal congestion, diarrhea, nausea or vomiting or |
| Pneumonia | Presence of pneumonia but no signs of severe pneumonia and no need for supplemental oxygen.  CURB≤1. |
| Severe pneumonia | Confirmed respiratory infection, plus one of the following:   1. Respiratory rate > 30 breaths/min. 2. Severe respiratory distress. 3. SpO2 ≤ 93% on room air. |
| Acute respiratory distress syndrome (ARDS) | **Onset:** within 1 week of a known clinical insult or new or worsening respiratory symptoms. **Chest imaging** (radiograph, CT scan, or lung ultrasound): bilateral opacities, not fully explained by volume overload, lobar or lung collapse, or nodules. **Origin of pulmonary infiltrates**: respiratory failure not fully explained by cardiac failure or fluid overload. Need objective assessment (e.g. echocardiography) to exclude hydrostatic cause of infiltrates/oedema if no risk factor present. **Oxygenation impairment in adults**:   - Mild ARDS: 200 mmHg < PaO2/FiO2a ≤ 300 mmHg (with PEEP or CPAP ≥ 5 cmH2O, or non-ventilated) - Moderate ARDS: 100 mmHg < PaO2/FiO2 ≤ 200 mmHg (with PEEP ≥ 5 cmH2O, or non-ventilated) - Severe ARDS: PaO2/FiO2 ≤ 100 mmHg (with PEEP ≥ 5 cmH2O, or non-ventilated) - When PaO2 is not available, SpO2/FiO2 ≤ 315 implies ARDS (including in non-ventilated patients). |

*Sp: Saturation percentage. ADRS: Acute Distress Respiratory Syndrome. CT: Cranial Tomography. PaO2: Partial pressure of Oxygen. FiO2: Fraction of inspired Oxygen. PEEP: Positive end-expiratory pressure. CPAP: Continuous positive airway pressure.*

1. Metlay JP, Waterer GW, Long AC, et al. Diagnosis and treatment of adults with community-acquired pneumonia: an official clinical practice guideline of the American Thoracic Society and Infectious Disease Society of America. *Am J Respir Crit Care Med* 2019; **200:** e45-e67.

**Sample size and power calculation:**

We estimated the power based on the mortality of the group with neurological symptoms on presentation (58/320, 18.1%) and the group without neurological symptoms (69/256, 27%) of our sample. Given an alpha error of 5%, using a bilateral type of test, for a sample size of 570 patients, the power would be 95%.

**Supplementary table 2:** Type, frequency and percentage of Covid-19 symptoms. In patients with non-unspecific neurological symptoms on admission compared with the rest of the sample.

| Variable | All patients (n=576) | Non-unspecific neurological symptoms on admission (n=100) | Rest of the sample  (n=476) | Adjusted p-value |
| --- | --- | --- | --- | --- |
| Arthralgia | 35 (6.1%) | 5 (5.0%) | 30 (6.3%) | 0.818 |
| Asthenia | 242 (42.0%) | 36 (36.0%) | 206 (43.3%) | 0.220 |
| Headache | 137 (23.8%) | 22 (22.0%) | 115 (24.2%) | 0.700 |
| Weakness | 90 (15.7%) | 23 (23.0%) | 67 (14.1%) | 0.033 |
| Diarrhoea | 192 (33.4%) | 25 (25.0%) | 167 (35.1%) | 0.062 |
| Dyspnoea | 292 (50.8%) | 46 (46.0%) | 246 (51.7%) | 0.323 |
| Chest pain | 99 (17.2%) | 10 (10.0%) | 89 (18.7%) | 0.041 |
| Expectoration | 90 (15.7%) | 14 (15.6%) | 76 (16.0%) | 0.762 |
| Fever | 462 (80.3%) | 72 (72.0%) | 390 (81.9%) | 0.027 |
| Anosmia | 146 (25.3%) | 14 (14.0%) | 132 (27.7%) | 0.004 |
| Light-headedness | 60 (10.4%) | 29 (29.0%) | 31 (6.5%) | <0.001 |
| Myalgia | 139 (24.1%) | 20 (20.0%) | 119 (25.0%) | 0.307 |
| Odynophagia | 60 (10.4%) | 5 (5.0%) | 55 (11.6%) | 0.070 |
| Cutaneous rash | 11 (1.9%) | 1 (1.0%) | 10 (2.1%) | 0.699 |
| Rhinorrhoea | 12 (2.1%) | 0 (0%) | 12 (2.5%) | 0.237 |
| Cough | 403 (70.2%) | 56 (56.0%) | 347 (73.1%) | 0.001 |
| Vomiting | 47 (8.2%) | 9 (9.0%) | 38 (8.0%) | 0.691 |

**Supplementary table 3: Demographic variables, clinical presentation and laboratory abnormalities in patients with absence of general symptoms.**

|  | **Patient 1** | **Patient 2** | **Patient 3** | **Patient 4** |
| --- | --- | --- | --- | --- |
| **Sex** | Male | Male | Male | Male |
| **Age** | 42 | 78 | 83 | 87 |
| **Comorbidities** | Hypertension, diabetes, HIV | Hypertension, EPOC, insomnia | Hypertension, diabetes, coronary artery disease, cancer, prior stroke | Hypertension, smoker, coronary artery disease, EPOC, cancer, Alzheimer disease |
| **Source of contagion** | Unknown | Infected relative | Unknown | Nursing home |
| **Days between the onset of symptoms and the ER presentation** | 0 | 0 | 0 | 0 |
| **Presenting symptom** | Loss of consciousness | Loss of consciousness | Altered mental status | Loss of consciousness |
|  | Increased LDH, Impaired renal function, increased D-dimer, increased CRP, increased PCT | Impaired renal function, increased D-dimer | Anemia, impaired renal function, increased D-dimer, increased CRP | Lymphopenia, increased LDH, Impaired renal function, increased D-dimer, increased CRP |

**Supplementary table 4:** **Sensitivity of general symptoms in Covid-19 patients with neurological symptoms on admission.**

|  | Sensitivity | 95% Confidence Interval |
| --- | --- | --- |
| Arthralgia | 10.3% | 7.3-14.3% |
| Asthenia | 46.2% | 40.7-51.8% |
| Weakness | 18.1% | 14.1-22.9% |
| Diarrhea | 35.3% | 30.1-40.8% |
| Dyspnea | 50.3% | 44.7-55.9% |
| Chest pain | 18.1% | 14.1-22.9% |
| Expectoration | 17.5% | 13.6-22.2% |
| Fever | 83.7% | 79.1-87.5% |
| Lightheadedness | 14.7% | 11.1-19.1% |
| Odynophagia | 11.2% | 8.1-15.3% |
| Cutaneous rash | 10.3% | 7.3-14.3% |
| Rhinorrhea | 46.2% | 40.7-51.8% |
| Cough | 75.0% | 69.8-79.6% |
| Vomiting | 35.3% | 30.1-40.8% |

**Supplementary table 5: Frequency of laboratory abnormalities in the whole sample and in patients with and without neurological symptoms on admission.**

| Variable | All patients (n=576) | Neurological symptoms on admission (n=320) | No neurological symptoms on admission (n=256) | Adjusted p-value |
| --- | --- | --- | --- | --- |
| Leukocytes  (RV: 4-10)  Units: count x 10^9^/L (n=574) | 167  (29.1%). | 91  (28.4%) | 76  (29.7%) | 0.813 |
| Lymphocytes (RV: >0.9)  Units: count x 10^9^/L (n=576) | 244  (42.4%) | 140  (43.8%) | 104  (40.6%) | 0.503 |
| Hemoglobin  (RV: >12)  Units: count x 10^9^/L (n=574) | 119  (20.7%) | 61  (19.1%) | 58  (22.7%) | 0.340 |
| Platelets  (RV: 150-400)  Units: count x 10^9^/L (n=557) | 147  (26.4%) | 88  (27.5%) | 59  (23.0%) | 0.262 |
| LDH  (RV: >250)  Units: U/L (n=563) | 362  (64.3%) | 187  (58.4%) | 175  (68.4%) | 0.018 |
| Glomerular filtration rate (corrected by body are)  (RV: >90)  Units: ml/min/1.73m^3^  (n=553) | 386  (69.8%) | 201  (62.8%) | 185 (72.3%) | 0.021 |
| Abnormal liver enzymes (AST <32, ALT <33, or GGT <40)  Units: U/L | 213  (39.3%) | 122  (39.6%) | 91  (38.9%) | 0.935 |
| INR  (RV: <1.3)  (n=561) | 131  (23.4%) | 63  (19.7%) | 68  (26.6%) | 0.063 |
| D-dimer  (RV: <500)  Units: ng/dL  (n=544) | 390  (71.7%) | 209  (65.3%) | 181  (70.7%) | 0.199 |
| Creatine-kinase  (RV: >170)  Units: U/L  (n=349) | 49  (21.6%) | 25  (7.8%) | 24  (9.4%) | 0.605 |
| C-reactive protein  (RV: < 5)  Units: mg/L  (n=564) | 520  (92.2%) | 284  (88.8%) | 236  (92.2%) | 0.214 |
| Procalcitonin  (RV: <0.5)  Units: ng/mL  (n=467) | 67  (14.3%) | 35  (52.2%) | 32  (12.5%) | 0.652 |

*RV: Reference value. U: Units. LDH: Lactate dehydrogenase, AST: aspartate aminotransferase, ALT: alanine aminotransferase; GGT: gamma-glutamyl transferase, INR: International normalized ratio.*

**Supplementary table 6: Sensitivity of laboratory parameters in Covid-19 patients with neurological symptoms on admission.**

|  | Sensitivity | 95% Confidence interval |
| --- | --- | --- |
| Leukocytes | 28.4% | 23.6-33.7% |
| Lymphocytes | 43.7% | 38.3-49.3% |
| Hb | 19.1% | 14.9-23.9% |
| Platelets | 27.5% | 22.7-32.8% |
| LDH | 58.4% | 52.8-63.8% |
| GFR | 62.8% | 57.2-68.1% |
| Liver enzymes | 39.6% | 34.1-45.3% |
| INR | 19.7% | 15.6-24.6% |
| D-dimer | 65.3% | 59.8-70.4% |
| CRP | 88.7% | 84.6-91.8% |
| PCT | 10.9% | 7.8-15.0% |

*Hb: hemoglobin, LDH: Lactate dehydrogenase, GFR: Glomerular Filtration Rate, INR: International normalized ratio. CRP: C-reactive protein. PCT: Procalcitonin.*

**Supplementary figure 1: Sensitivity (and 95% confidence interval) of general symptoms and laboratory parameters in Covid-19 patients with neurological symptoms on admission.**


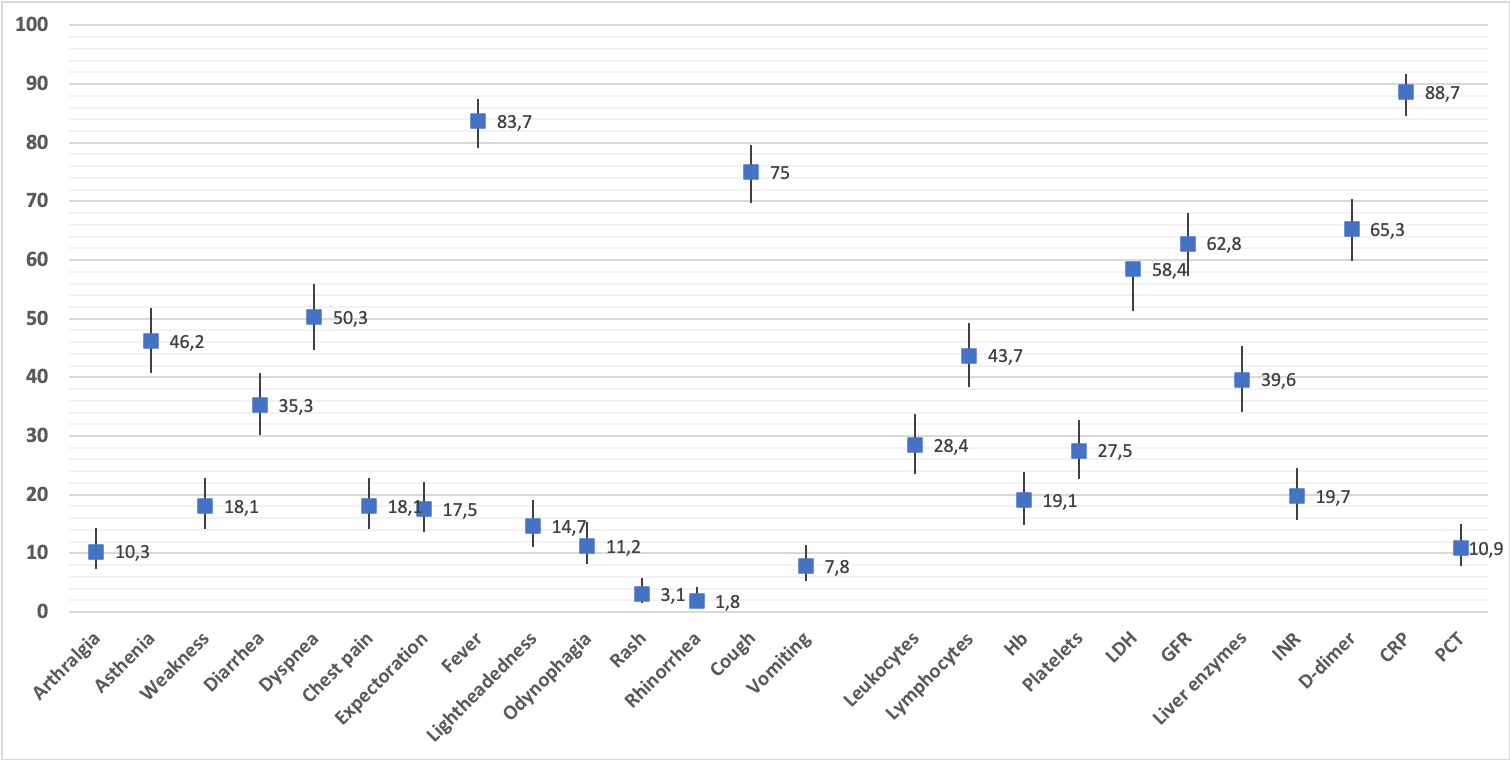
*Hb: hemoglobin, LDH: Lactate dehydrogenase, GFR: Glomerular Filtration Rate, INR: International normalized ratio. CRP: C-reactive protein. PCT: Procalcitonin.*

**Supplementary table 7: Predictors of mortality: Univariate Cox-regression analysis:**

| Variable | Exp(B) | 95% CI | P value |
| --- | --- | --- | --- |
| Female sex | 0.859 | 0.579-1.275 | 0.452 |
| Age | 1.079 | 1.060-1.098 | <0.001 |
| mRS3 | 7.572 | 5.059-11.333 | <0.001 |
| Time since symptoms’ onset | 0.888 | 0.858-0.919 | <0.001 |
| Hypertension | 3.239 | 2.079-5.045 | <0.001 |
| Diabetes | 1.940 | 1.285-2.929 | 0.002 |
| Smoking | 1.380 | 0.900-2.116 | 0.140 |
| Cardiological disorders | 2.358 | 1.601-3.474 | <0.001 |
| Pulmonary disorders | 1.188 | 0.778-1.815 | 0.426 |
| Cancer | 1.300 | 0.805-2.097 | 0.283 |
| Immunosuppression | 2.024 | 1.083-3.780 | 0.027 |
| Chronic neurological disorders | 4.106 | 2.781-6.063 | <0.001 |
| Headache at onset | 0.246 | 0.120-0.506 | <0.001 |
| Anosmia at onset | 0.142 | 0.058-0.347 | <0.001 |
| Myalgia at onset | 0.415 | 0.237-0.729 | 0.002 |
| Syncope at onset | 1.414 | 0.736-2.716 | 0.298 |
| Altered mental status | 3.261 | 2.132-4.987 | <0.001 |
| Arthralgia | 0.263 | 0.065-1.066 | 0.061 |
| Asthenia | 0.409 | 0.266-0.631 | <0.001 |
| Weakness | 0.807 | 0.467-1.395 | 0.443 |
| Diarrhea | 0.642 | 0.412-0.999 | 0.050 |
| Dyspnea | 1.621 | 1.079-2.437 | 0.020 |
| Chest pain | 0.430 | 0.224-0.826 | 0.011 |
| Expectoration | 0.860 | 0.505-1.464 | 0.578 |
| Fever | 0.450 | 0.297-0.682 | <0.001 |
| Lightheadedness | 0.948 | 0.529-1.700 | 0.859 |
| Rash | 0.048 | 0.000-29.250 | 0.354 |
| Odynophagia | 0.082 | 0.011-0.590 | 0.013 |
| Rhinorrhea | 0.047 | 0.000-9.174 | 0.256 |
| Cough | 0.425 | 0.289-0.625 | <0.001 |
| Vomiting | 0.625 | 0.274-1.426 | 0.264 |

*mRS: Modified Rankin scale. CI: Confidence interval.*

**Supplementary table 8: Predictors of mortality: Univariate logistic regression analysis:**

| Variable | OR | 95% CI | P value |
| --- | --- | --- | --- |
| Female sex | 0.654 | 0.454-1.024 | 0.065 |
| Age | 1.090 | 1.069-1.112 | <0.001 |
| mRS3 | 11.371 | 6.376-20.278 | <0.001 |
| Time since symptoms onset | 0.908 | 0.870-0.947 | <0.001 |
| Hypertension | 3.534 | 2.272-5.495 | <0.001 |
| Diabetes | 2.129 | 1.353-3.351 | 0.001 |
| Smoking | 1.589 | 1.004-2.514 | 0.048 |
| Cardiological disorders | 2.955 | 1.950-4.478 | <0.001 |
| Pulmonary disorders | 1.434 | 0.928-2.217 | 0.105 |
| Cancer | 1.641 | 1.001-2.690 | 0.049 |
| Immunosuppression or chemotherapy | 2.238 | 1.063-4.713 | 0.034 |
| Chronic neurological disorders | 3.961 | 2.516-6.234 | <0.001 |
| Headache at onset | 0.193 | 0.091-0.407 | <0.001 |
| Anosmia at onset | 0.103 | 0.041-0.257 | <0.001 |
| Myalgia at onset | 0.429 | 0.243-0.755 | 0.003 |
| Syncope at onset | 1.543 | 0.740-3.215 | 0.247 |
| Altered mental status | 4.500 | 2.675-7.570 | <0.001 |
| Arthralgia | 0.202 | 0.048-0.852 | 0.029 |
| Asthenia | 0.403 | 0.260-0.625 | <0.001 |
| Weakness | 0.796 | 0.451-1.406 | 0.432 |
| Diarrhea | 0.578 | 0.369-0.905 | 0.016 |
| Dyspnea | 2.263 | 1.500-3.416 | <0.001 |
| Chest pain | 0.388 | 0.200-0.751 | 0.005 |
| Expectoration | 0.937 | 0.541-1.623 | 0.815 |
| Fever | 0.407 | 0.260-0.638 | <0.001 |
| Lightheadedness | 1.085 | 0.576-2.045 | 0.800 |
| Odynophagia | 0.228 | 0.081-0.642 | 0.005 |
| Rhinorrhea | 0.326 | 0.040-2.471 | 0.272 |
| Cough | 0.435 | 0.289-0.656 | <0.001 |
| Vomiting | 0.707 | 0.322-1.554 | 0.388 |

*mRS: Modified Rankin scale. CI: Confidence interval.*

**Supplementary table 9: Predictors of mortality: Multivariate Cox-regression analysis:**

| Variable | Exp(B) | 95% CI | P value |
| --- | --- | --- | --- |
| Female sex | 0.957 | 0.595-1.540 | 0.856 |
| Age | 1.047 | 1.022-1.072 | <0.001 |
| mRS3 | 2.029 | 1.172-3.512 | 0.011 |
| Time since symptoms onset | 0.946 | 0.913-0.981 | 0.003 |
| Hypertension | 1.367 | 0.830-2.250 | 0.219 |
| Diabetes | 1.097 | 0.693-1.736 | 0.694 |
| Smoking | 1.315 | 0.789-2.189 | 0.293 |
| Cardiological disorders | 1.021 | 0.667-1.564 | 0.923 |
| Immunosuppression | 1.083 | 0.384-1.472 | 0.88 |
| Chronic neurological disorders | 2.250 | 1.446-3.502 | <0.001 |
| Headache at onset | 0.679 | 0.314-1.472 | 0.327 |
| Anosmia at onset | 0.358 | 0.140-0.916 | 0.032 |
| Myalgia at onset | 0.872 | 0.457-1.666 | 0.679 |
| Altered mental status | 1.867 | 1.162-3.001 | 0.010 |
| Arthralgia | 0.557 | 0.12-2.598 | 0.456 |
| Asthenia | 0.420 | 0.262-0.671 | <0.001 |
| Diarrhea | 0.940 | 0.580-1.524 | 0.802 |
| Dyspnea | 2.636 | 1.642-4.231 | <0.001 |
| Chest pain | 0.547 | 0.275-1.088 | 0.086 |
| Fever | 0.765 | 0.481-1.217 | 0.258 |
| Odynophagia | 0.191 | 0.026-1.401 | 0.103 |
| Cough | 0.857 | 0.535-1.372 | 0.520 |

*mRS: Modified Rankin scale. CI: Confidence interval.*

**Supplementary table 10: Predictors of mortality: Multivariate Cox-regression analysis (restrictive analysis):**

| Variable | Exp(B) | 95% CI | P value |
| --- | --- | --- | --- |
| Age | 1.047 | 1.023-1.072 | <0.001 |
| mRS3 | 1.922 | 1.023-3.268 | 0.016 |
| Time since symptoms onset | 0.943 | 0.908-0.979 | 0.002 |
| Hypertension | 1.312 | 0.798-2.157 | 0.285 |
| Diabetes | 1.111 | 0.710-1.739 | 0.644 |
| Cardiological disorders | 1.111 | 0.730-1.564 | 0.623 |
| Immunosuppression | 1.552 | 0.789-3.389 | 0.203 |
| Chronic neurological disorders | 2.175 | 1.305-3.389 | 0.001 |
| Headache at onset | 0.665 | 0.310-1.430 | 0.297 |
| Anosmia at onset | 0.335 | 0.131-0.875 | 0.022 |
| Myalgia at onset | 0.877 | 0.466-1.650 | 0.685 |
| Altered mental status | 1.911 | 1.192-3.064 | 0.007 |
| Arthralgia | 0.639 | 0.138-2.957 | 0.567 |
| Asthenia | 0.387 | 0.240-0.623 | <0.001 |
| Diarrhea | 0.885 | 0.549-1.427 | 0.617 |
| Dyspnea | 2.772 | 1.737-4.423 | <0.001 |
| Chest pain | 0.508 | 0.255-1.011 | 0.054 |
| Fever | 0.731 | 0.459-1.403 | 0.187 |
| Cough | 0.872 | 0.541-1.403 | 0.571 |

*mRS: Modified Rankin scale. CI: Confidence interval.*

**Supplementary table 11: Predictors of mortality: Multivariate logistic regression analysis:**

| Variable | OR | 95% CI | P value |
| --- | --- | --- | --- |
| Female sex | 0.621 | 0.340-1.135 | 0.122 |
| Age | 1.064 | 1.036-1.093 | <0.001 |
| mRS3 | 2.647 | 1.209-5.795 | 0.015 |
| Time since symptoms onset | 0.976 | 0.936-1.018 | 0.262 |
| Hypertension | 1.109 | 0.608-2.022 | 0.735 |
| Diabetes | 1.179 | 0.630-2.206 | 0.607 |
| Smoking | 1.367 | 0.697-2.681 | 0.362 |
| Cardiological disorders | 1.343 | 0.762-2.366 | 0.307 |
| Pulmonary disorders | 0.646 | 0.347-1.205 | 0.17 |
| Cancer | 1.231 | 0.615-2.463 | 0.558 |
| Immunosuppression or chemotherapy | 2.492 | 0.894-6.946 | 0.081 |
| Chronic neurological disorders | 1.576 | 0.845-6.946 | 0.153 |
| Headache at onset | 0.569 | 0.225-1.437 | 0.233 |
| Anosmia at onset | 0.166 | 0.056-0.491 | 0.001 |
| Myalgia at onset | 1.743 | 0.783-3.880 | 0.174 |
| Altered mental status | 3.296 | 1.62-6.708 | 0.001 |
| Arthralgia | 0.277 | 0.047-1.630 | 0.156 |
| Asthenia | 0.423 | 0.237-0.756 | 0.004 |
| Diarrhea | 1.008 | 0.555-1.831 | 0.978 |
| Dyspnea | 4.542 | 2.489-8.287 | <0.001 |
| Chest pain | 0.483 | 0.208-1.121 | 0.09 |
| Fever | 0.634 | 0.346-1.164 | 0.141 |
| Odynophagia | 0.337 | 0.096-1.180 | 0.089 |
| Cough | 0.893 | 0.488-1.635 | 0.715 |

*mRS: Modified Rankin scale. CI: Confidence interval.*

**Supplementary table 12: Predictors of mortality: Multivariate logistic regression analysis (restrictive analysis):**

| Variable | OR | 95% CI | P value |
| --- | --- | --- | --- |
| Female sex | 0.645 | 0.355-1.173 | 0.151 |
| Age | 1.062 | 1.034-1.090 | <0.001 |
| mRS3 | 2.679 | 1.224-5.862 | 0.014 |
| Time since symptoms onset | 0.980 | 0.940-1.022 | 0.343 |
| Hypertension | 1.140 | 0.627-2.071 | 0.668 |
| Diabetes | 1.154 | 0.620-2.148 | 0.652 |
| Smoking | 1.212 | 0.631-2.326 | 0.564 |
| Cardiological disorders | 1.287 | 0.733-2.258 | 0.380 |
| Cancer | 1.219 | 0.609-2.440 | 0.576 |
| Immunosuppression or chemotherapy | 2.411 | 0.879-6.607 | 0.087 |
| Chronic neurological disorders | 1.555 | 0.834-2.896 | 0.165 |
| Headache at onset | 0.558 | 0.221-1.411 | 0.218 |
| Anosmia at onset | 0.178 | 0.061-0.520 | 0.002 |
| Myalgia at onset | 1.651 | 0.743-3.668 | 0.218 |
| Altered mental status | 3.315 | 1.629-6.745 | 0.001 |
| Arthralgia | 0.317 | 0.055-1.830 | 0.199 |
| Asthenia | 0.419 | 0.235-0.749 | 0.003 |
| Diarrhea | 1.050 | 0.581-1.898 | 0.871 |
| Dyspnea | 4.272 | 2.369-7.702 | <0.001 |
| Chest pain | 0.472 | 0.203-1.096 | 0.081 |
| Fever | 0.645 | 0.353-1.180 | 0.155 |
| Odynophagia | 0.336 | 0.095-1.191 | 0.091 |
| Cough | 0.877 | 0.481-1.600 | 0.669 |

*mRS: Modified Rankin scale. CI: Confidence interval.*
